# Perinatal outcome among fasting and non fasting mothers during the month of Ramadan

**DOI:** 10.12669/pjms.344.15654

**Published:** 2018

**Authors:** Zahe Gul, Seema Rajar, Zeenat Fareed Shaikh, Kashif Shafique, Nazli Hossain

**Affiliations:** 1 Dr. Zahe Gul, MBBS. Resident Medical Officer, Department of Obstetrics & Gynecology Unit-II, Dow Medical College & Dr. Ruth K.M. Pfau Civil Hospital, Karachi, Pakistan; 2 Dr. Seema Rajar, MBBS. Resident Medical Officer, Department of Obstetrics & Gynecology Unit-II, Dow Medical College & Dr. Ruth K.M. Pfau Civil Hospital, Karachi, Pakistan; 3 Dr. Zeenat Fareed Shaikh, MBBS, FCPS. Consultant Gynecologist, Department of Obstetrics & Gynecology Unit-II, Dow Medical College & Dr. Ruth K.M. Pfau Civil Hospital, Karachi, Pakistan; 4 Dr. Kashif Shafique, MBBS, MPH, PhD. Honorary Clinical Senior Lecturer (University of Glasgow), Principal - School of Public Health, Head - Department of Research, Dow University of Health Sciences, Karachi, Pakistan; 5 Prof. Dr. Nazli Hossain, MBBS, FCPS, MBE. Department of Obstetrics & Gynecology Unit-II, Dow Medical College & Dr. Ruth K.M. Pfau Civil Hospital, Karachi, Pakistan

**Keywords:** Ramadan, Maternal fasting, Pakistan, Placental weight

## Abstract

**Objective:**

To compare the perinatal outcome among fasting and non fasting pregnant mothers.

**Methods:**

A total of 180 women, who came for delivery in the labor suite were included, after verbal informed consent. These women were divided in two groups fasting (n=100), and non-fasting (n=80).

**Results:**

The mean age of the mothers was 27.16±4.27 years in the fasting group and 27.36±4.92 years in non-fasting group. The average BMI of mothers was 25.31±3.26 kg/m^2^in fasting group while 25.64±3.58 kg/m^2^in non-fasting group. Perinatal outcomes, the birth weight, head circumference and mid arm circumference were almost similar between the two groups. Weight of placenta was 537.80±80.01g in fasting group while 540.50±84.29 g in non-fasting group and height of baby was 45.79±3.07 cm in fasting group while 46.61±2.92 cm in non-fasting group. In fasting group, placenta weight was 531.5±92.80 g in boys while 544.8±62.79 g in girls and ratio of placental to birth weight was 18.8±2.28 in boys while 19.4±2.70 in girls.

**Conclusion:**

Maternal fasting affects placental weight and length of baby, with effect more pronounced in male babies.

## INTRODUCTION

Ramadan is the holy month in the Muslim calendar, in which abstinence from food and drink is observed from sunrise to sunset. The duration of fast varies from region to region, depending on the geographic location. The last Ramadan month started from May 28, 2017 and ended by June 28, 2017 in Pakistan. The average duration of fast was around 15 hours and few minutes.

Fasting is considered as one of the five pillars of Islam, and is mandatory to fast. Exemptions are granted for sick, elderly, pregnant and nursing mothers. Though pregnant mothers are exempted from fasting, it is observed that most of them do fast during the month of Ramadan, for social reasons and for their own convenience. Women are supposed to complete the count of days, whenever they consider it feasible. The number of women who will fast while being pregnant will vary from region to region. For example in Iran, around 70% of pregnant women will fast during the month of Ramadan, whereas in England and Singapore this has been reported to be around 90%.^1^ In a cross sectional study from our region, including more than 300 women, 88% of the women thought fast during pregnancy was obligatory, in the presence of good health, whereas 12% thought otherwise.^2^

There are few studies on the impact of fasting on pregnancy. An Iranian cohort of 189 women (both fasting and non fasting) did not show any effect on pregnancy outcome, including anthropometric measures of newborn.^3^ Moradi M conducted a study comparing growth parameters including femoral length, bi parietal diameter, amniotic fluid volume among fasting and non fasting pregnant women, and did not find any statistical difference between the two groups.^4^ In another study from a country where the Islamic month was observed during summer season (July-August), investigators found a decrease in the amniotic fluid volume among fasting pregnant mothers, when compared to non fasting mothers.^5^ Karateke A et al., in a cohort of more than 200 women, also did not find any significant difference in the growth parameters of fetus among fasting and non fasting women, except increase in amniotic fluid and maternal weight gain amongst women who fasted during second trimester.^6^ In another prospective cohort study from Netherlands, women who fasted during the first trimester had lighter weight newborns, compared to non fasting women (-198 g, 95% CI -447, 51, P= 0·12), but this was not found statistically significant.^1^ In this study, women with light weight newborns had fasted for a minimum of 20 days, during the month of Ramadan.

In a prospective cohort study of more than 400 women, fasting was found not to be associated with length of gestation. The preterm delivery rate among fasting versus non fasting women (10.4% versus 10.4%).^7^ But the authors did find decreased birth weight among fasting women when compared to non-fasting (3202±473 gm versus 3094±467 gm P=0.024). Another cross sectional study from Iran, including 4000 pregnant women, also did not find maternal fasting to influence birth weight of newborn.^8^

It is a well-known fact that fetus draws its nutrition from the placenta in a large cohort from Saudi Arabia, placental weight and ratio of placental weight with birth weight and gender was calculated for more than 4000 births during the study period, and was compared with those who did not give birth during the fasting month. The investigators found that mean placental weight and ratio of placental weight to birth weight was lower in the newborns, whose mother fasted.^9^ And this was also affected by the gender of the baby. Placental weight was affected more in boys, compared to girls.

We do not have such studies from our part of the region, where fasts are longer, and climate is harsher, with high temperatures. A PubMed search did not reveal any such study exploring the effects of fasting from South-Asian region. The aim of this study was to determine if maternal fasting affects fetal growth parameters at birth, including placental weight.

## METHODS

This study was conducted in the department of Obstetrics & Gynecology Unit-II, Dr. Ruth K.M. Pfau Civil Hospital and Dow Medical College. The study period was from July 2017 to December 2017.

Women admitted in the labor room, for delivery were included, after informed verbal consent. Women were divided in two groups, those who fasted during Ramadan, and those who did not fast during the month of Ramadan. The fasting period was divided into three periods, viz first trimester, second trimester and third trimester. Women were included in the fasting group, if they had fasted for a minimum of 10 days, and had minimum of three booking visits. Women with known systemic disorders like essential hypertension, insulin dependent diabetes, gestational and type 2 diabetes mellitus, any other chronic medical illness or those with congenital anamolus baby, intrauterine demise were excluded from the study. Also excluded were non booked women and those diagnosed with intrauterine growth restriction and multiple pregnancies.

A random-purposive sampling technique was used for data collection on a predesigned questionnaire. It included demographic details, days of fasting, maternal weight gain during pregnancy, gestational age, delivery details and anthroprometric measurements of newborn (weight in kilograms, height (cms), length (cms) and mid arm circumference (cm) of baby. Placental weight including cord and membranes was measured, within one hour of delivery. Institutional review board of the University approved the study.

### Statistical analysis

**Fig.1:**
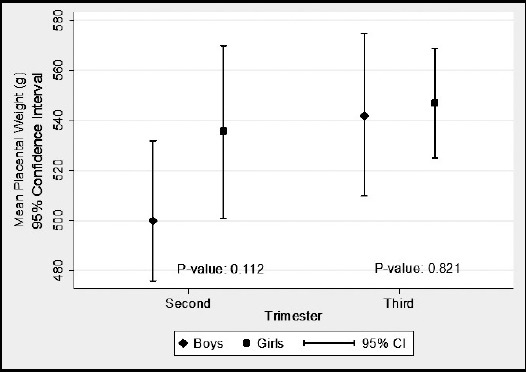
Mean placental weight (g) according to trimester in fasting group.

**Fig.2:**
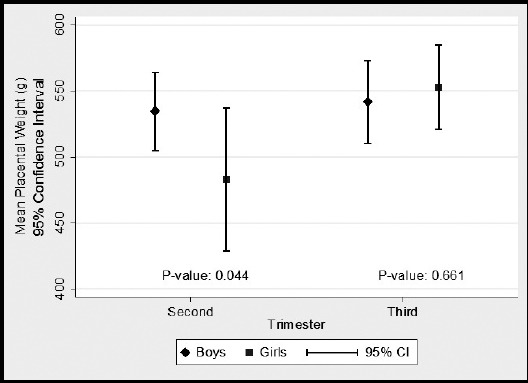
Mean placental weight in non fasting group.

Data were analyzed by using SPSS version 16.0. Descriptive statistics for perinatal outcomes were reported as mean and standard deviation, while frequencies and percentages were reported for all categorical characteristics. Pearson chi-square test was used to examine the association between fasting and non-fasting groups according to maternal characteristics and socio demographics. For continuous characteristics like maternal age, parity, body mass index (BMI), neonatal birth weight and height, weight of placenta, head circumference, mid arm circumference, associations were assessed between both groups by using Independent sample t-test. Further, gender wise differences among perinatal outcomes between fasting and non-fasting groups were also assessed using Independent sample t-test. (Fig. 1 & 2) A value of p<0.05 was considered significant.

## RESULTS

A total of 180 pregnant women with their birth record were included in the study. Of these, 100 women were in fasting group while 80 were in non-fasting group. The mean age of the mothers was 27.16±4.27 years in the fasting group and 27.36±4.92 years in non-fasting group. The average BMI of mothers was 25.31±3.26 kg/m^2^in fasting group while 25.64±3.58 kg/m^2^in non-fasting group. Among fasting group, 24% (n=24) were in second trimester, and 76% (n=76) were in third trimester as compared to non-fasting group where 20% (n=16) were in second trimester and 80% (n=64) were in third trimester. In fasting group, 4% (n=4) were preterm delivery and 96% (n=96) were term delivery as compared to non-fasting group where 2.5% (n=2) were preterm delivery and 97.5% (n=78) were term deliveries. There were no significant differences in maternal age, parity, maternal BMI, number of trimester and gestational length between fasting and non-fasting groups (Table-I).

**Table-I:** Comparison of maternal characteristics between the fasting and non-fasting group. (n=180).

Characteristics	Fasting Group n=100	Non-Fasting Group n=80	
	Mean±SD	Mean±SD	p-value*
Age (years)	27.16±4.27	27.36±4.92	0.768
Parity	3.23±1.54	3.04±1.59	0.414
BMI (kg/m^2^)	25.31±3.26	25.64±3.58	0.511
	n(%)	n(%)	p-value*
**Type of Trimester**			
Second	24 (24.0)	16 (20.0)	0.521
Third	76 (76.0)	64 (80.0)	
**Gestational Length**			
Preterm Delivery	4 (4.0)	2 (2.5)	0.694
Term Delivery	96 (96.0)	78 (97.5)	

* P-value calculated by using Independent sample t- test and Chi-square test

There was no significant differences between mother’s socio-demographic characteristic and fasting status as shown in Table-II. Regarding perinatal outcomes, the birth weight, head circumference and mid arm circumference were almost similar between the two groups. Weight of placenta was 537.80±80.01g in fasting group while 540.50±84.29 g in non-fasting group and height of baby was 45.79±3.07 cm in fasting group while 46.61±2.92 cm in non-fasting group which indicates that weight of placenta and height of baby were slightly higher in non-fasting group as compared to fasting group. (Table-III).

**Table-II:** Comparison of sociodemographic characteristics between the fasting and non-fasting group (n=180).

Characteristics	Fasting Group n=100 n (%)	Non-Fasting Group n=80 n (%)	p-value*
**Gender of newborn**			
Male	53 (53.0)	49 (61.2)	0.267
Female	47 (47.0)	31 (38.8)	
**Ethnicity**			
Urdu	29 (29.0)	29 (36.3)	0.125
Sindhi	21 (21.0)	21 (26.3)	
Balochi	14 (14.0)	6(7.5)	
Pathan	27 (27.0)	11 (13.7)	
Punjabi	5 (5.0)	7 (8.7)	
Others	4 (4.0)	6(7.5)	
**Educational Status**			
Illiterate	42 (42.0)	36 (45.0)	0.425
Primary	25 (25.0)	12 (15.0)	
Secondary	12 (12.0)	10 (12.5)	
Matric	16 (16.0)	14 (17.5)	
**Intermediate/Graduate**	5 (5.0)	8 (10.0)	

* P-value calculated by using Chi-square test

**Table-III:** Comparison of neonates and perinatal outcome between the fasting and non-fasting group (n=180).

Characteristics	Fasting Group n=100 Mean±SD	Non-Fasting Group n=80 Mean±SD	p-value*
Birth weight (kg)	2.82±0.38	2.86±0.41	0.549
Height of Baby (cm)	45.79±3.07	46.61±2.92	0.071
Head Circumference (cm)	33.77±1.51	33.72±1.29	0.802
Mid Arm Circumference (cm)	10.56±1.05	10.32±0.88	0.119
Weight of Placenta (g)	537.80±80.01	540.50±84.29	0.826

* P-value calculated by using Independent sample t-test.

Perinatal outcomes remained similar when the analysis was stratified based on the gender of the babies. The birth weight, height of baby, head circumference and mid arm circumference were almost similar in fasting and non-fasting groups. For fasting group, placenta weight was 531.5±92.80 g in boys while 544.8±62.79g in girls and ratio of placental to birth weight was 18.8±2.28 in boys while 19.4±2.70 in girls which indicates that placenta weight and ratio of placental to birth weight were slightly higher in girls as compared to boys. Similarly, the differences in birth weight, placenta weight, height of baby, head circumference and mid arm circumference were almost similar in non-fasting group. Ratio of placental weight to birth weight was 19.1±2.39 in boys while 18.7±2.20 in girls which indicates that ratio of placental to birth weight was slightly higher in boys as compared to girls. Results showed no significant differences in perinatal outcomes based on gender of babies in fasting and non-fasting groups (Table-IV).

**Table-IV:** Comparison of perinatal outcome on the basis of gender (n=180).

Characteristics	Fasting Group		p-value*	Non-Fasting Group		p-value*
	Boys n=53	Girls n=47		Boys n=49	Girls n=31	
	Mean±SD	Mean±SD		Mean±SD	Mean±SD	
Birth weight (kg)	2.8±0.45	2.8±0.28	0.802	2.8±0.41	2.8±0.40	0.631
Placenta Weight (g)	531.5±92.80	544.8±62.79	0.407	540.8±88.78	540±78.10	0.967
Placental/birth weight ratio (%)	18.8±2.28	19.4±2.70	0.215	19.1±2.39	18.7±2.20	0.530
Height of Baby (cm)	45.7±3.25	45.8±2.88	0.823	46.8±2.90	46.2±2.96	0.343
Head Circumference (cm)	33.5±1.71	33.9±1.22	0.207	33.7±1.25	33.7±1.38	0.945
Mid Arm Circumference (cm)	10.5±1.21	10.6±0.85	0.723	10.2±0.89	10.4±0.87	0.535

* P-value calculated by using Independent sample t-test.

In fasting group, mean placenta weight was slightly higher in girls (536±47.88g) as compared to boys (500±55.47g) in second trimester while mean placenta weight was slightly higher in girls (547±66.11g) as compared to boys (542±101.17g) in third trimester but there were no significant differences in placenta weight according to gender in second and third trimester. Fig.1. Among non-fasting group, mean placenta weight was higher in boys (535±41.6g) as compared to girls (483±51.84g) in second trimester which shows that there was significant differences in placenta weight according to gender in second trimester (P-value <0.04) while mean placenta weight was higher in girls (553±77.9g) as compared to boys (542±97.6g) in third trimester which shows that there were no significant differences (P-value <0.66) in placenta weight according to gender in third trimester. Fig.2

## DISCUSSION

The main aim of the study was to see whether maternal fasting affects neonatal growth parameters and placental weight. The demographic characteristics of both groups of women were similar in our study. (Table-I) The mean body mass index of women in both groups was 25, which is above the normal cut-off for Asian women.^10^,^11^ The socioeconomic backgrounds for both groups were similar, as both belonged to low socio economic class. This was a single center study with uniformity in the study population. The weight of the placenta has been shown to be affected by body mass index. Roland MC et al. have shown in a study of more than 1000 Scandinavian women, an increase in a unit of BMI resulted in an increase of 0.79gm of placental weight.^12^ This increase in placental weight was also associated with gestational weight gain, in early trimesters of pregnancy. The effect of body mass index on placental weight was also seen in an Iranian study which observed that maternal fasting resulted in increase in the birth weight, when compared to non-fasting mothers.^13^ But this was attributed to increased body mass index of fasting mothers. In our study BMI of women in both groups were similar. We did not have record of gestational weight gain; hence cannot comment upon whether weight of placenta has any association with gestational weight gain.

In our study placental weight and ratio of placental weight to birth weight was found to be decreased in women who fasted. Weight of placenta was 537.80±80.01g in fasting group while 540.50±84.29 g in non-fasting group and height of baby was 45.79±3.07 cm in fasting group while 46.61±2.92 cm in non-fasting group which indicates that weight of placenta and height of baby were slightly higher in non-fasting group as compared to fasting group. This decrease in placental weight was more pronounced in boys. Gender based influence on placental weight has been observed in earlier studies, with placental weights more in boys as compared to girls.^14^ Placenta not only acts as conduit to transport nutrition from maternal side to fetus, its syncytiotrophoblast also secretes hormones into maternal circulation, which in turn facilitates fetal growth.^15^ The total placental area available for exchange of nutrients is 11m^2^at term. Decreased placental weight have been observed in small for gestational age babies.^16^ In this study the investigators compared placental weight of appropriate for gestational age new born (n=15047) with small for gestational age new born (n=1549). The latter group was found to have smaller placenta, leading to the conclusion that fetal growth is dependent upon weight of placenta. Whether this change in the placental weight have any influence on adult programming of the disease?^17^ This is the area of future research.^17^

In our study, majority of the mothers fasted in the third trimester. We did not find any difference in the birth weight of newborns between the two groups (2.82±0.38 vs. 2.86±0.41 p-value 0.549). In another prospective study by Karateke A, fetal birth weight was not found to be affected by maternal fasting.^6^ In a prospective study of 130 women from Netherlands, maternal fasting during first trimester was associated with light weight newborns (-272g, 95% CI -547, 3, P= 0.05).^1^ In our study population, we did not have women who fasted in first trimester. This is logical to explain as first trimester is associated with nausea and vomiting, making it difficult for women to fast during first trimester in tropical and hot areas.

### Limitations of the study

The sample size is small to generalize the results of the study. We also did not take into account gestational weight gain due to logistic reasons. The uniformity in demographics in study population, adds strength to the study. Moreover, this is first study from Pakistan looking at the perinatal outcomes, hence providing some idea to obstetrician and clinician in providing advice to fasting pregnant women.

### Author’s Contribution

**ZG and SR** assisted in data collection.

**ZS** assisted in compiling data collection.

**KS** assisted in data analysis.

**NH** conceived the idea, executed it and prepared the manuscript.
